# 

**Published:** 1871-05

**Authors:** 


### CONTENTS:

### ORIGINAL COMMUNICATIONS.

ARTICLE I.—Alloying and Refining Gold for Dental Purposes. By Mintern J. Brown, D.D.S. ..... 1

ARTICLE II.—Cleft Palate. By Wm. J. Fogle, D.D.S. ..... 8

ARTICLE III.—Proceedings of Georgia State Dental Association. By R. A. McDonald, D.D.S. 10

ARTICLE IV.—Alabama Dental Association. By Dr. Robert A. Savage ..... 14

### SELECTED ARTICLES.

ARTICLE V.—Case of Triple Fracture of the Lower Jaw. By T. Curtis Smith, M. D. ..... 15

ARTICLE VI —Caries and Necrosis ..... 16

ARTICLE VII.—Preparation of Chloral By E. R. Squibb, M. D. ..... 24

ARTICLE VIII.—Quick Method of Making Dies. By J. A. Troutman ..... 30

ARTICLE IX.—Excision of Inferior Maxillary Nerve ..... 31

ARTICLE X.—Neuralgia of Jaw following Tooth Extraction ..... 39

### MONTHLY SUMMARY.

Carbolic Acid Preparations ..... 40

Oil of Peppermint as an Anæsthetic ..... 41

California Quicksilver ..... 41

Mixing Plaster Impressions ..... 42

Treating Discolored Teeth ..... 42

To Polish Marble ..... 42

Hydrate of Chloral ..... 43

Death from Chloroform ..... 43

Bleaching Beeswax ..... 44

Death from Inhalation of Ether ..... 44

Cancrum Oris ..... 44

### EDITORIAL DEPARTMENT.

Dental Items.—Calcified Pulp ..... 45
Swallowing Artificial Teeth ..... 46
Treating of Soft Tissues of the Mouth ..... 47
Heavy Gold Foil ..... 48
Alveolar Abscess ..... 48

